# Mobile DNA elements in T4 and related phages

**DOI:** 10.1186/1743-422X-7-290

**Published:** 2010-10-28

**Authors:** David R Edgell, Ewan A Gibb, Marlene Belfort

**Affiliations:** 1 Department of Biochemistry, Schulich School of Medicine & Dentistry, The University of Western Ontario, London, ON, N6A 5C1, Canada; 2 Wadsworth Center, New York State Department of Health, Center for Medical Sciences, 150 New Scotland Ave., Albany, NY 12208, USA

## Abstract

Mobile genetic elements are common inhabitants of virtually every genome where they can exert profound influences on genome structure and function in addition to promoting their own spread within and between genomes. Phage T4 and related phage have long served as a model system for understanding the molecular mechanisms by which a certain class of mobile DNA, homing endonucleases, promote their spread. Homing endonucleases are site-specific DNA endonucleases that initiate mobility by introducing double-strand breaks at defined positions in genomes lacking the endonuclease gene, stimulating repair and recombination pathways that mobilize the endonuclease coding region. In phage T4, homing endonucleases were first discovered as encoded within the self-splicing *td*, *nrdB *and *nrdD *introns of T4. Genomic data has revealed that homing endonucleases are extremely widespread in T-even-like phage, as evidenced by the astounding fact that ~11% of the T4 genome encodes homing endonuclease genes, with most of them located outside of self-splicing introns. Detailed studies of the mobile *td *intron and its encoded endonuclease, I-TevI, have laid the foundation for genetic, biochemical and structural aspects that regulate the mobility process, and more recently have provided insights into regulation of homing endonuclease function. Here, we summarize the current state of knowledge regarding T4-encoded homing endonucleases, with particular emphasis on the *td*/I-TevI model system. We also discuss recent progress in the biology of free-standing endonucleases, and present areas of future research for this fascinating class of mobile genetic elements.

## Introduction

**Figure 1:**
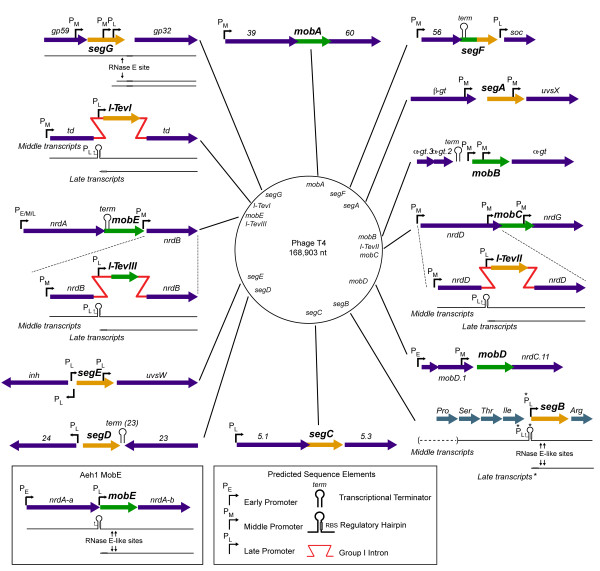
**Schematic of the location of the fifteen homing endonuclease genes indicated on a genomic map of bacteriophage T4**. For simplicity, each genomic segment is drawn with the endonuclease in the same orientation, with relevant regulatory elements indicated. The GIY-YIG endonucleases are shown in yellow, while the HNH-type endonucleases are green. The hybrid endonuclease *segF *is drawn with both colours. The bacteriophage Aeh1 *mobE *endonuclease, which is not part of the bacteriophage T4 genome, is set in a box. An asterix (*) marks a predicted late promoter upstream of SegB.

In the 20 years since the first review on mobile genetic elements in the T4 genome, significant progress has been made with respect to understanding the biology of T4-encoded homing endonucleases [1]. In particular, we now have a firm grasp of the DNA repair and recombination pathways that promote mobility of intron-encoded endonucleases [2-5]. We also know more about the molecular details that regulate protein-DNA interactions of the long-serving model homing endonuclease, I-TevI, providing intriguing insights into how the enzyme has adapted well to life in a genome rich in glucosylated hydroxymethylcytosine-containing DNA [6-8]. Perhaps one of the most surprising discoveries was the finding that T4 encodes 12 homing endonucleases that are not intron encoded, but instead are located in intergenic regions (Figure 1, Table 1). The so-called free-standing endonucleases belong to the GIY-YIG and HNH homing endonuclease families, and are termed *seg *(similar to endonucleases encoded within group I introns) and *mob *(mobility) genes, respectively [9,10]. In recent years, the explosion of phage genome sequences has revealed that free-standing endonucleases are more widespread than their intron-encoded cousins (at least in T-even phage genomes), while at the same time confirming a long-held suspicion that T4 is an oddity among T-even-like phages, for no other phage comes close to encoding the 15 homing endonucleases that T4 does - representing 11% of its coding potential!

**Table 1:** The homing endonucleases of phage T4

Endonuclease	Intron encoded or free-standing	Active	Family	Insertion Site	Target Gene	Reference
I-TevI	Intron	Yes	GIY-YIG	*td*	*td*	[14,28]
I-TevII	Intron	Yes	GIY-YIG	*nrdD*	*nrdD*	[7]
I-TevIII	Intron	Yes (RB3)	HNH	*nrdB*	*nrdB*	[19,20]
*mobA*	Free-standing	ND	HNH	*60.1*/*39*	*39*	D. Shub pers. comm..
*mobB*	Free-standing	ND	HNH	*α-gt*/*α-gt.2*	unknown	
*mobC*	Free-standing	ND	HNH	*nrdG*/*nrdD*	unknown	
*mobD*	Free-standing	ND	HNH	*nrdC.11*/*mobD.1*	unknown	
*mobE*	Free-standing	Yes	HNH	*nrdB*/*nrdA*	*nrdB*	[41,42]
*segA*	Free-standing	Yes	GIY-YIG	*uvsX*/*β-gt*	*uvsX*	[34]
*segB*	Free-standing	Yes	GIY-YIG	tRNA-Arg/tRNA-Ile	tRNA intergenic region	[38]
*segC*	Free-standing	Yes	GIY-YIG	*5.1*/*5.3*	of *5.1 *and *5.3*	[37]
*segD*	Free-standing	ND	GIY-YIG	*23*/*24*	unknown	
*segE*	Free-standing	Yes	GIY-YIG	*inh*/*uvsW*	*uvsW*	[36]
*segF*	Free-standing	Yes	GIY-YIG	*soc*/*56*	*56*	[35]
*segG*	Free-standing	Yes	GIY-YIG	*32*/*59*	*32*	[39]

Our purpose in this review is to summarize the past 20 years of research into T4 homing endonucleases, with emphasis on the mechanisms involved in mobility, protein-DNA recognition, and the regulation of endonuclease function within the context of a host genome. Because mechanistic insights into endonuclease function stemming from studies on T4-encoded endonucleases will be generally applicable to endonucleases encoded within other T-even phage genomes, we will focus mainly on T4 endonucleases, discussing examples in other phage only when obvious differences are found. We also point out areas for future research where we are still largely ignorant, namely the mobility pathways utilized by the *mob *endonucleases, which nick rather than cleave their targets, transcriptional and translational regulation of endonucleases, and questions of an evolutionary nature dealing with the impact of endonuclease activity on phage genome structure and function.

## Mechanisms of mobility

### Pathways

The variable occurrence of the three T4 introns in other closely related T-even phage first suggested that these introns, in the *td*, *nrdB*, and *nrdD *(*sunY*) genes, are mobile genetic elements [11,12]. Shortly after these observations, mobility was demonstrated for the *td *and *nrdD *introns, and attributed to intron-encoded endonucleases that make a double-strand break (DSB) [13]. The first mechanistic insight came from the observation that intron insertion into the cleaved target, the so-called homing site, is accompanied by co-conversion of the flanking exon sequences [14]. Cleavage of target DNA by an intron endonuclease and co-conversion of flanking exon sequences are both features associated with mobile introns of eukaryotes [15], indicating a common mechanism for intron transfer. Indeed in both cases co-conversion of exon markers reflects the DSB being processed to a gap [16].

**Figure 2:**
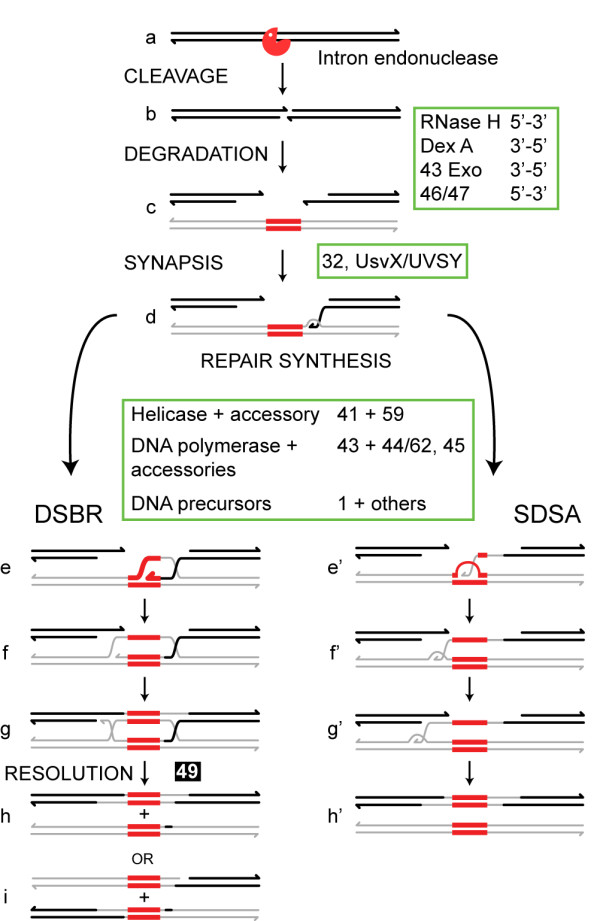
**Alternative mechanisms for DSB-mediated intron homing**. Subsequent to cleavage by the homing endonuclease (a), the recipient, intronless allele (thick lines) undergoes exonucleolytic degradation and homologous sequence alignment with an intron-containing donor (thin lines) (b, c). A 3' end of the recipient invades the donor, which serves as a template for repair synthesis (d). In the DSBR pathway (left), DNA synthesis through the intron (red) results in formation and expansion of a D-loop (e), which then serves as substrate for repair synthesis of the noninvading strand (f). Holliday junctions are resolved to produce either noncrossover (h) or crossover (i) products. During synthesis-dependent strand annealing (SDSA) (right), the displaced loop or bubble migrates with the replicative end as DNA synthesis proceeds through the intron (e' - g'). The newly synthesized strand is released from the donor and serves as template for repair synthesis of the noninvading strand (g' - h') to generate noncrossover products only (h'). Functions implicated in homing and their putative association with appropriate steps in the homing pathways are shown.

Because of the facile phage/bacterial genetic system, *td *intron homing has the best characterized group I intron inheritance pathway. Key studies involved defining both bacterial and phage functions that are required for the homing event as well as characterizing recombination intermediates [3,17]. Mobility depends on host or phage recombinase functions, RecA or UvsX, respectively. The process also uses phage-encoded exonuclease activities, single-stranded binding proteins (Gp32), DNA synthesis and repair functions, resolvase and ligase (Figure 2). In light of these dependencies, and exon co-conversion, it was concluded that introduction of the DSB is followed by exonucleolytic degradation [18], and that the processed 3' end invades the intron donor duplex and primes repair synthesis that results in copying of the intron into the recipient DNA. This process likely proceeds for at least some events via the DSB repair (DSBR) pathway, wherein a D-loop formed as the result of repair synthesis serves as a template for repair of the opposite strand [3,16] (Figure 2, left pathway). The two Holliday junctions formed during the repair process can be resolved to yield two intron-containing alleles: if the junction is cleaved in the crossover orientation flanking markers are exchanged, whereas if the junction is cleaved in the non-crossover orientation no exchange of flanking markers is observed. The T4 gene *49 *product resolves these junctions [3].

Interestingly, homing is reduced but not abolished in gene *49 *mutants. This ambiguous requirement for gp49 implies alternative resolution enzymes or additional homing pathways. The underrepresentation of crossover events among the homing products favors alternative pathway(s), of which the synthesis-dependent strand annealing (SDSA) pathway is one (Figure 2, right pathway). The initial steps of the SDSA pathway are the same as those of DSBR, but unlike DSBR, Holliday junctions are not formed, circumventing the need for resolvase and resulting only in non-crossover products [3].

There is a close relationship between intron mobility and recombination-dependent replication in phage T4 [3]. Thus, intron homing occurs in mutants in which origin-dependent replication is disrupted [primase (gp61) and topoisomerase (gp39, gp52, gp60)], but is reduced in recombination-dependent replication mutants. The latter functions that play a role in homing include recombinase activities (UvsX, UvsY), single-stranded DNA-binding protein (gp32), exonucleolytic functions (RNaseH, DexA, 43Exo and possibly gp46, gp47), DNA polymerase (gp43) and its accessories (gp44, gp45, and gp62), helicase (gp41), the primase-helicase accessory (gp59), enzymes providing DNA precursors (eg. gp1) and DNA ligase (gp30) (Figure 2).

T4 RNase H, a 5'-3' exonuclease, T4 DNA exonuclease A (DexA) and the 3'-5' exonuclease activity of T4 DNA polymerase (*43*Exo) impact not only degradation, but also the homing efficiency and flanking marker coconversion [2]. The experiments implicating a role for these functions in intron homing provided the first direct evidence of a role for 3' ssDNA tails in T4 recombination. Together, the work that defined the involvement of phage accessories to the homing process demonstrates how a mobile intron harnesses phage replication, recombination and repair functions for its own propagation [2,3,17].

Although the above discussion is based on homology between donor and recipient, heterologous sequences can also participate in DSB-mediated repair [4,5]. Extensive homology in one exon supports elevated homing levels when the other exon is absent, allowing analysis of "one-sided" events, which revealed illegitimate DSB repair. Recombination junctions at sites of microhomology and extensive nucleolytic degradation were evident. These observations suggest that illegitimate DSB repair may provide a means by which introns can invade ectopic sites, while lengthy resection may also be related to distal cleavage sites of the freestanding endonucleases, to be considered below.

### Proteins: intron-encoded endonucleases

The intron-encoded endonucleases of the T-even phage genome are members of the GIY-YIG and HNH families. These families are characterized based on the catalytic cleavage domains, which are joined to DNA binding domains of varying specificities. The phage T4 *td*, *nrdB*, *nrdD *introns encode, respectively, the following endonucleases: I-TevI and I-TevII, both GIY-YIG endonucleases, and I-TevIII, a member of the HNH family. I-TevIII is, however, inactive on account of a large deletion, but a functional ortholog is found in phage RB3 [19,20]. The DNA-binding domains of both the phage-encoded endonuclease families appear to be architectually similar, in a beads-on-a-string arrangement, consisting of a variety of small protein modules that gives the proteins their specificity [21,22].

**Figure 3:**
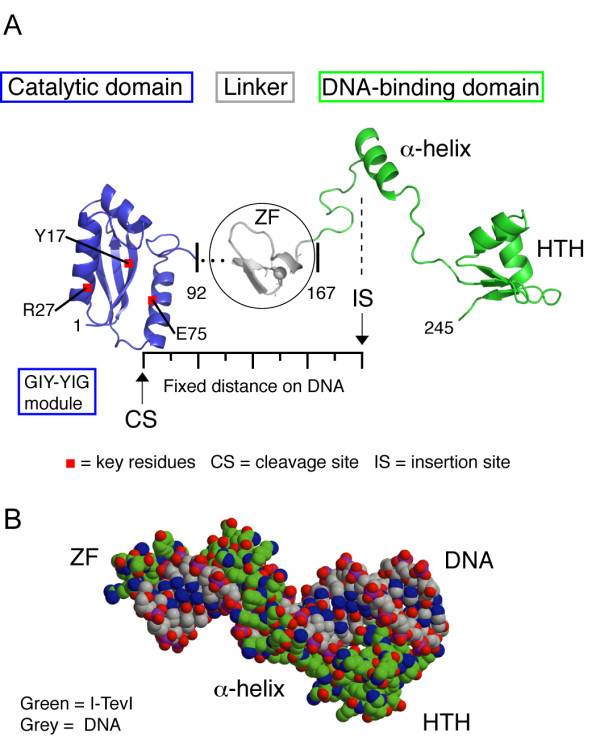
**I-TevI structure**. A. Two domains of the enzyme joined by a linker. The catalytic GIY-YIG domain (blue) is separated from the DNA binding domain (green) by a 75-amino acid linker, which includes the zinc finger (grey). The DNA binding domain consists of elongated segments, an α-helix and a helix-turn-helix (HTH) module. B. Space filling model of the DNA-binding domain and zinc finger on DNA. The protein is bound to a 20-bp DNA substrate.

The best characterized of the T-even phage enzymes is the GIY-YIG *td *intron endonuclease I-TevI (Figure 3). The GIY-YIG family of endonucleases was first identified as representing sequence similarities in intron-encoded proteins of phage T4 and filmentous fungi [23]. Now, more than 20 years later, we know, mainly from multiple sequence alignments, of a large GIY-YIG superfamily of enzymes that nicks or cleaves DNA. This superfamily encompasses restriction enzymes, retrotransposons, and recombination and repair proteins, including UvrC, which performs nucleotide excision repair [24].

I-TevI and I-TevII, both GIY-YIG endonucleases, have several features in common. First, they have lengthy recognition sequences, spanning more than two helical turns of DNA; second, they induce conformational changes in the homing site during the substrate binding and cleavage process; third, they bind in the minor groove; and, finally they remain bound to the cleaved substrate [6,7,25]. Minor-groove binding is easily reconciled with T4 DNA being heavily modified in the major groove. Persistent binding to the cleavage product is also more than a curiosity, accounting for exon coconversion asymmetry. I-TevI, for example, remains bound to the exon II side of the homing site, resulting in coconversion biases in exon I, which is free for digestion by degradative nucleases [18].

The GIY-YIG module in I-TevI is 92 amino long, has five conserved motifs, of which GIY-N_10/11_-YIG is the first (Motif A) and is thought to play a structural role. Motifs B, D and E contain conserved Arg, Glu and Asn residues, which function in catalysis [26]. The catalytic domain is joined to a lengthy, and distinct DNA-binding domain, which recognizes an expansive target sequence [6], [27]. The 28-kDa I-TevI recognizes a 38-bp target sequence, binding as a monomer. I-TevI cleaves intronless DNA at sites 23 nt and 25 nt upstream of the intron insertion site (IS) to create a DSB, but how a monomeric enzyme cleaves two strands is not known [21]. In constrast, the HNH endonuclease I-TevIII of phage RB3, which is structurally and catalytically intact, acts as a dimer to make a DSB [20].

The monomeric I-TevI interacts with two regions of its 38-bp homing site [28]. The DNA-binding domain, which has an extended structure, winds around the primary binding region of 20 bp, centered on the intron IS [8] (Figure 3). This domain is joined via a long linker to the globular GIY-YIG-containing catalytic domain, which contacts the cleavage site (CS). The linker is 75-amino acids long, and has elements of structure, including a C-terminal zinc finger, which abuts the DNA-binding domain [29]. This linker is responsible for dynamic properties of I-TevI, and facilitates a dual role, namely to act as both an endonuclease or a transcriptional autorepressor [30,31].

**Figure 4:**
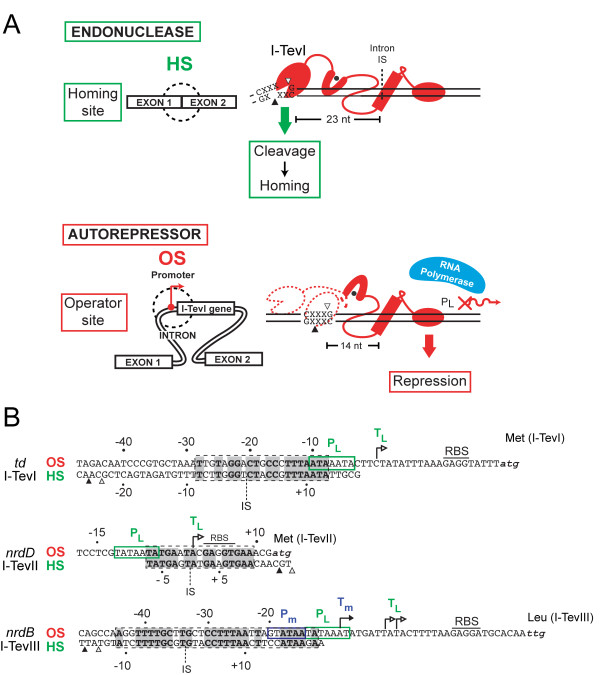
**Dual function of I-TevI**. A. I-TevI binds with equal affinity to the homing site (top) and operator site (bottom). The CS sequence at the natural distance in the homing site allows endonuclease cleavage, to initiate homing. In the operator site, there is no cleavage sequence at a suitable distance, resulting in autorepression, because I-TevI binding blocks the late promoter and transcription. B. Autorepression by T4 intron-encoded endonucleases. For each of the three endonucleases, I-TevI, I-TevII, and I-TevIII, the endonuclease's homing site (HS) is aligned with proven or putative the operator site (OS) upstream of the endonuclease ORF within the *td*, *nrdB*, and *nrdD *introns, respectively. The operator sites are indicated by dashed boxes, with bold-type nucleotides representing identity between the operator and homing sites. The position of the endonuclease's cleavage sites are indicated by open and black triangles. Green and blue boxes indicate late and middle T4 promoters, respectively, with corresponding transcription start sites indicated by right-facing arrows labeled with the same color.

I-TevI uses both sequence and distance determinants in selecting its CS [25]. Although the enzyme is generally tolerant of nucleotide changes in the homing site [6], it has a preference for both its natural cleavage sequence, and for the wild-type distance. If its CS is displaced from the optimal distance of 23 nt and 25 nt, I-TevI searches bidirectionally from its cleavage position to locate a preferred site, 5'-CX↑XX↓G-3', and cleaves at alternative distances, albeit with reduced efficiency [25,30,32]. The cleavage window extends from 5 bp upstream to 16 bp downstream of the normal cleavage site [25]. When a preferred site is not within the window, the enzyme defaults to the optimal distance and cleaves with reduced efficiency [25,32]. Most of the linker (except for ~20 N-terminal amino acids adjacent to the catalytic domain) and the zinc finger, serves as the distance determinant to constrain the catalytic domain, such that it is proximal to the cleavage site and promotes catalysis [29,31,32]. One of the functions of the linker is therefore to act as a "protein ruler", which we postulated to have evolved because I-TevI moonlights as an autorepressor, as described in section 3 [29-31]. Thus, the overall role of the I-TevI linker is to act as a communication device between the DNA-binding and catalytic GIY-YIG domains, such that they act in concert for DNA cleavage, but the DNA-binding domain acts independently when serving as a transcriptional repressor (Figure 4A) [31].

The zinc finger imparts another layer of regulation to I-TevI cleavage. In addition to the structural diversification that zinc fingers provide, an interesting aspect of these modules is their ability to be regulated by oxidation and reduction (redox) reactions [33]. Indeed, the zinc finger of I-TevI is redox responsive, and acts as a switch altering the ability of the enzyme to faithfully cleave its cognate substrate (Robbins, Smith and Belfort, in preparation). Under reducing conditions, the zinc finger is intact, active and accurate, whereas upon oxidation, the zinc is lost, and I-TevI suffers comprised activity and fidelity. We speculate that oxidative stress may provide a signal to the enzyme, transduced via the zinc finger, to cleave at ectopic sites, and thereby to facilitate intron spread.

### Proteins: Free-standing endonucleases

Of the 15 homing endonucleases encoded in the T4 genome, 12 are free standing and found in the intergenic regions separating genes that are conserved amongst related phage genomes (Figure 1). Early work showed that SegA is a site-specific DNA endonuclease that generates a DSB with a 2-nucleotide 3' extension in the *uvsX *gene, consistent with its similarity to intron-encoded GIY-YIG endonucleases, which also generate 2-nt 3' extension [34]. Subsequent work demonstrated activity for a number of *seg *endonucleases (Table 1) [35-39]. Furthermore, *seg *endonucleases are inherited at high frequencies in the progeny of T4 and T2 mixed infections, showing that homing endonucleases could be mobile elements outside of a host group I intron or intein. The term intronless homing was coined to distinguish mobility of free-standing endonucleases from intron-encoded versions [35], and to emphasize one striking difference - the position of the enzyme's CS's relative to the insertion site of the endonuclease gene. Unlike known intron-encoded endonucleases, which cleave within 25 bps of the intron insertion site, the CS of free-standing endonucleases are located hundreds or thousands of base pairs distant from the endonuclease gene. This separation of cleavage and insertion sites has important consequences for inheritance of a free-standing endonuclease, because exonucleolytic ressection of the DSB in the recipient genome must extend into regions of homology flanking the free-standing endonuclease in the donor genome in order to ensure that it is inherited in progeny phage [40]. Furthermore, sequencing of co-conversion tracts associated with mobility events of a number of free-standing endonucleases are consistent with DSB repair pathways [39,41].

Compared to the *seg *endonucleases, comparatively little is known about the five *mob *endonucleases of phage T4. Each of the *mob *genes consists of a well-defined HNH nuclease domain fused to a distinct C-terminal region, presumably the DNA-binding domain. Consistent with the presence of an HNH domain, both MobA and MobE nick one strand of their target substrates, and are inherited at high frequency in progeny of mixed infections [41,42]. Similar observations were made for I-HmuI and I-HmuII, HNH endonucleases encoded within group I introns of *Bacillus *phage [43-45]. One outstanding question regarding the HNH endonucleases is how does the introduction of a single-strand nick promote a recombination event. It is possible that nicks are converted to a recombinogenic DSB by collapse of a passing replication fork [46], or by subsequent processing of the nick by repair enzymes. However, persistent DSBs associated with endonuclease-generated nicks could not be detected by Southern blot analyses [43].

## Regulation of homing endonuclease function

### Transcriptional Regulation - Promoter choice

One potential impact on phage viability from an invading endonuclease stems from disruption of a coding sequence and another from perturbing the expression of host genes that neighbor the endonuclease insertion site by displacing existing promoters upon insertion. This is not a trivial concern, as many T4 promoters are located in intergenic regions, precisely the insertion sites of free-standing endonucleases [47]. Alternatively, an invading endonuclease can introduce additional promoters that enhance transcription of neighboring genes, or create antisense transcripts if the promoter is placed in the opposite transcriptional orientation to surrounding genes. Thus, in order to persist in the phage population, an invading homing endonuclease must successfully integrate into the host transcriptional program to minimize the impact on surrounding host genes.

The regulatory elements that govern expression of the three T4 intron-encoded endonucleases I-TevI, I-TevII, and I-TevIII were deduced soon after the discovery of the introns and endonucleases themselves [48]. Primer extension analyses located the middle and late promoters that drive expression of the three endonucleases, with the common theme that these transcripts are embedded within early or middle transcriptional units of the interrupted *td*, *nrdD, *and *nrdB *genes (Figure 1). In contrast, seven free-standing endonucleases appear to be promoter-less cassettes. In these cases, for *segC*, *segF*, *segG*, *mobA*, *mobC*, *mobD*, and *mobE*, the endonucleases harness upstream T4 promoters to become part of an existing polycistronic message. Because insertion of some of the *seg *and *mob *endonucleases displaced existing T4 transcription starts, promoters for downstream genes are embedded within the endonuclease's coding regions. For instance, the middle and late promoters that drive expression of the essential gene *32 *are positioned within *segG *(formerly *32.1*) [49], with similar cases found for *segA*, *segE*, *mobB*, and *mobC*. This arrangement of embedded promoters favors retention of the endonuclease in the phage genome, because any deletion event that removed the essential host promoter would be detrimental to the phage.

Seven free-standing endonuclease genes (with one exception, *segD*) are all transcribed from promoters that lie in the non-coding regions upstream of the endonuclease ORF (*segB, segE, *and *mobB)*, or in the 3' region of the gene immediately upstream of the endonuclease (*segA, segG, mobC *and *mobD)*. Interestingly, these "endonuclease-specific" promoters are either middle (5 instances) or late (2 instances), with no occurrences of early promoters, suggesting that there is some advantage to expression of endonucleases >5 min post infection.

All of the homing endonuclease genes in T4 are present in the same transcriptional orientation as the surrounding genes, with one notable exception. The uncharacterized free-standing GIY-YIG endonuclease *segD *is oriented in the opposite transcriptional direction to the surrounding genes *23 *and *24*, encoding the essential major capsid protein and vertex protein, respectively (Figure 1). This arrangement of *segD *with respect to the surrounding T4 genes is noteworthy in that bioinformatic searches failed to identify a *segD*-specific promoter. Thus, *segD *expression may depend on transcription events that initiate at either the late promoter upstream of the *inh *gene (~4.8 kb from *segD*) or one of two middle promoters upstream of or internal to *24.2 *(~2.9 kb and ~2.4 kb from *segD*, respectively). Yet, transcripts initiated from these promoters would have to read through intrinsic transcriptional terminators up- and downstream of *segD*. Given the antisense orientation of *segD *and extensive transcriptional terminator in this region of the T4 genome, it is not unreasonable to assume that *segD *transcript levels are vanishingly low.

### Intron-encoded endonucleases also function as transcriptional autorepressors

An added layer of transcriptional regulation was recently discovered for I-TevI [30]. In examining DNA sequence immediately upstream of the I-TevI ORF, strong similarity (15/20 nucleotide identity) was observed between a sequence that overlapped the late promoter that drives expression of I-TevI and the I-TevI homing site (Figure 4A &4B). A similar arrangement was also observed for I-TevII and I-TevIII, whereby potential binding sites with similarity to the endonuclease's homing site overlapped the middle or late promoters upstream of I-TevII and I-TevIII (Figure 4B). This arrangement of binding sites (operators) and promoters suggested that each of the T4 intron endonucleases also functioned as transcriptional autorepressors, regulating their expression by binding to operator sites to occlude the middle and late promoters from RNA polymerase. Indeed, I-TevI was shown to bind its operator site with the same affinity as its homing site, and functioned to downregulate expression of *lacZ *fused to the I-TevI late promoter during phage infection. Although not experimentally demonstrated for I-TevII or I-TevIII, it is likely that each endonuclease also functions like I-TevI to autoregulate its own expression.

One immediate question raised by the finding of an operator site was whether I-TevI cleaved the operator site with similar efficiency as its homing site, as I-TevI bound with similar affinity to the operator and homing sites. However, cleavage assays showed that I-TevI cleaved the operator site ~100-fold less efficiently than the homing site [30]. Reduction in I-TevI cleavage efficiency can be attributed to the lack of a critical 5'-CXXXG-3' sequence positioned appropriately to the I-TevI operator site (as described in section 2B). Interestingly, zinc finger mutants of I-TevI cleave the operator site more efficiently than the homing site substrate [31]. Zinc finger mutants, which have lost the ability to constrain cleavage to a fixed distance, can scan for a sub-optimally placed 5'-CXXXG-3' sequence, which in the case of operator substrate lies at positions that would be equivalent to -15 through -19 of the homing site. Thus, the I-TevI zinc finger possesses two biological functions - to ensure that the enzyme cleaves at the optimal distance on homing site substrate to promote intron homing, and to prevent cleavage on the operator substrate to promote persistence of the *td *intron and I-TevI in the phage population (Figure 4).

### Regulation by transcript processing

Transcriptional termination mediated by intrinsic or rho-independent terminators plays a key role in regulating the expression of T4 genes, and many intrinsic terminators have been computationally identified [47]. T4 terminators are very similar to *E. coli *intrinsic terminators, characterized by a GC-rich stem, a 4-nucleotide loop, and a poly(U) tract immediately downstream of the stem structure [50-52]. Interestingly, two free-standing endonucleases, *mobE *and *segF*, possess intrinsic transcriptional terminators in the 5' end of their coding regions [47,53]. The *mobE *endonuclease is inserted in the *nrdA*/*nrdB *intergenic region of a number of T-even phage, and its expression is dependent on promoters upstream of *nrdA*. The terminator internal to *mobE *was predicted to be weak based on the length of the poly(U) tract [54], and RNase protection assays and mapping of 3' ends have shown that ~30% of transcripts terminate at the poly(U) tract that immediately follows the *mobE *terminator [42]. However, transcription of the essential *nrdB *gene downstream of *mobE *is not affected by the presence of the terminator in *mobE*, because a middle promoter is located in the intergenic space separating the 3' end of *mobE *and 5' end of *nrdB *[53]. One potential biological function of the *mobE *terminator is to limit read-through transcription from the *nrdA *promoter, modulating transcript levels of *nrdB *to coordinate synthesis of NrdB (the small subunit of aerobic ribonucleotide reductase) with that of NrdA (the large subunit) [53,55]. More speculatively, the terminator may also be a T4-specific adaptation to regulate *mobE *expression, reducing the amount of *mobE*-containing transcripts.

Similarily, post-transcriptional processing of T4 *segB *and *segG *by the host enzyme RNase E may be an adaptation to reduce endonuclease transcript levels [38,56]. An RNase E-like processing site was also described in the *mobE *transcript in the T-even-like phage Aeh1 that infects *Aeromonas hydrophila *[57]. For T4 *segB *and Aeh1 *mobE*, the extent and timing of RNase E processing is unknown, while for *segG*, RNase E processing has been shown to increase the stability of the downstream gene *32*, facilitating translation [57]. It should be noted, however, that RNase E processing does not appear to affect the ability of *segG *or *segB *to act as mobile elements, as both endonucleases are inherited at high frequency in the progeny of T4 × T2 co-infections [38,39].

### Translational regulation - Involvement of RNA structures

**Figure 5:**
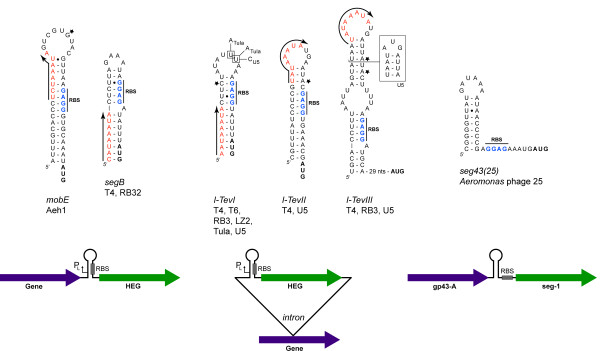
**RNA structures involved in translational regulation of homing endonucleases in T-even bacteriophage**. Arrows and red type indicate late promoter position and direction of transcription in the corresponding DNA sequence. For cases where the initating nucleotide has been mapped, it is indicated with a star. The RBS and start codon are shown in blue and black boldface type, respectively. Nucleotide variants in the I-TevI hairpin are indicated for phages TuIa and U5, and the alternative structure of the phage U5 I-TevIII hairpin is indicated by a box. The lower arrows indicate the general genetic organization of hairpin-regulated homing endonucleases. From left to right; the free-standing homing endonucleases *mobE *and *segB*, the intron-encoded endonucleases *I-TevI*, *I-TevII*, and *I-TevIII*, and the unusual hairpin for *seg43(25) *in *Aeromonas *phage 25.

The first hint that translational regulation was an important mechanism in the regulation of T4 homing endonucleases came from studies on the intron-encoded endonucleases I-TevI, I-TevII, and I-TevIII [48]. All three endonucleases possess a consensus Shine-Dalgarno sequence (or ribosome binding site, RBS) positioned approximately 8 nucleotides upstream of the AUG initation codon (Figure 5). However, a very stable RNA secondary structure sequesters the RBS such that translation would be very inefficient. For I-TevI, this RNA structure only forms on transcripts that initiate from early and middle promoters upstream of *td*, preventing translation of I-TevI at early and middle times during infection. A late promoter, immediately upstream of I-TevI, is positioned such that late transcripts do not include sufficient sequence to form the inhibitory RNA structure, freeing the RBS and facilitating translation of I-TevI at late times [30,48]. Similar arrangements of promoters and RNA secondary structures are found for I-TevII and I-TevIII, for T4 *segB*, and for *mobE *in phage Aeh1 (Figure 5) [30,38,48,57,58]. One departure from this mechanism of translational regulation is found for a *segD*-like endonuclease, *seg43(25)*, in *Aeromonas *phage 25 [59]. Here, a predicted RNA hairpin with very high stability folds immediately upstream of the RBS for *seg43(25)*, but the hairpin does not sequester the RBS that is immediately downstream from the base of the stem (Figure 5). It remains to be determined if this arrangement results in translational regulation.

Translational repression by sequestration of the RBS by an RNA structure is not unique to homing endonucleases, and has been shown for T4 genes including *soc*, *e*, *39*, *25 *and *26 *[60-63]. Interestingly, one commonality shared by homing endonucleases and T4 genes regulated by this mechanism is the fact that they are all late genes, and present on long polycistronic transcripts that encode early and middle gene products. Such translational regulation of late gene products may represent a mechanism to temporally orchestrate translation of gene products on polycistronic messages.

### Other potential translational regulation mechanisms

For the remaining free-standing homing endonucleases in phage T4, the translational control mechanisms are not obvious, and have yet to be addressed experimentally. The free-standing endonuclease ORFs start with an AUG initiation codon, the lone exception being *mobA *that is predicted to start at a GUG codon. In addition, translation initiation regions (TIRs) that are a reasonable match to the T4 consensus can be identified upstream of only six of the twelve endonuclease genes (for *segB*, *segC*, *segD*, *segE*, *mobA*, and *mobC*). However, the RBSs are not positioned at the optimal distance of 6-9 nucleotides from the AUG codon. For instance, the predicted *segD *RBS is 2 nucleotides upstream of the AUG codon, whereas the *mobC *RBS is 27 nucleotodies upstream, questioning whether or not these sequences represent *bone*-*fide *translation start sites.

Six of the twelve free-standing endonucleases have no discernable TIRs, with five of the endonuclease ORFs overlapping the upstream ORFs. In the case of *mobE*, the AUG iniation codon of *mobE *overlaps by one nucleotide with the first of two termination codons of the upstream *nrdA *gene, creating the following arrangement UAAUGA. This arrangement of overlapping initiation and termination codons is suggestive of translational coupling, a mechanism where termination of the upstream protein is linked to initiation of translation the downstream protein [64]. Such arrangements are thought to provide a mechanism to control the relative amounts of protein products that function in the same biological processes. For instance, translational coupling regulates the production of the clamp loader proteins gp46 and gp62 [65-67]. In most cases, translational coupling results in a lower relative amount of the downstream gene product to the upstream gene product, likely due to the reduced frequency of translation re-initiation at an internal AUG codon. Thus, if translational coupling is the mechanism by which MobE is expressed, the *nrdA*/*mobE *overlap may represent a mechanism to limit translation of MobE endonuclease, in addition to the aforementioned transcriptional termination in *mobE*.

In other cases, the extent of overlap with the upstream ORF is more extreme, as evidenced for the homing endonucleases *segC *(20-bp overlap), *segF *(11-bp overlap), *mobA *(23-bp overlap), and *mobC *(24-bp overlap). Here, the mechanism of translational control is likely to be ribosome 'scanning', whereby the ribosome does not dissociate and diffuses along the mRNA within a seven-codon window up- or down-stream of the termination codon [68]. If an AUG codon is encountered within this window, translation is initiated but at a low frequency, resulting in lower protein levels of the downstream endonuclease relative to the upstream protein.

### Why regulation?

Clearly, all of the mechanisms described above are negative regulatory mechanisms that function to downregulate the levels of homing endonucleases in T4-infected cells, suggesting that unregulated expression of endonucleases would be detrimental to T4. One obvious rationale for downregulating endonuclease function relates to the sequence-tolerant binding ability of homing endonucleases, and the potential for introducing (presumably) deleterious nicks and DSBs at ectopic sites throughout the T4 genome. Curiously, certain endonucleases, namely the intron-encoded endonucleases and *mobE *in phage Aeh1, are subject to multiple-layers of regulation, whereas other homing endonucleases are not as tightly regulated. Whether the intron-encoded endonucleases and *mobE *are more 'toxic' and require multi-layered regulation is an interesting possibility that requires experimental confirmation. Intriguingly, Kruezer and co-workers generated a T4 phage where the I-TevI operator site was deleted and replaced by a middle promoter, with no noticeable effect on phage viability [69]. Similarly, no endonuclease-mediated effect on phage viability was observed when the regulatory hairpin structure limiting translation of I-TevI to late in phage infection was deleted, facilitating translation of I-TevI at middle time points after phage infection [70].

However, recent work suggests that the stringent regulation of I-TevI is required to facilitate efficient splicing of the *td *intron and translation of full-length thymidylate synthase [70]. An interesting observation regarding I-TevI, and many other intron-encoded endonucleases, is that while the majority of the I-TevI ORF is located in a non-essential loop of the *td *intron, the 3' end of the ORF extends into the structured region of the intron and contributes key nucleotides that form critical secondary structures [71]. This observation led to the suggestion that translation of intron-encoded endonucleases from within the highly structured intron may interfere with intron folding and splicing [48]. Indeed, T4 phage mutant for the I-TevI translational regulatory hairpin exhibited a significant decrease in *td *intron splicing, an accumulation of unspliced *td *pre-mRNA, and a thymidine-dependent phenotype [70]. These observations suggest that one biological rationale for stringent, multi-layered regulation of intron-encoded endonucleases is a requirement to limit ribosome access to the intron core, ensuring proper splicing of the intron and function of the host gene interrupted by the intron [72,73].

One interesting commonality among all the regulatory mechanisms is the restriction of endonuclease function to middle or late times in the phage infective cycle [48,74]. We previously argued that such temporal regulation coordinates expression of the endonucleases with the DNA repair and replication machinery of phage T4, ensuring that the appropriate machinery and genome equivalents are present to repair endonuclease-mediated breaks and promote homing [30]. Delayed expression may also be a means to coincide homing endonuclease synthesis with recombination-dependent replication.

## Evolution of Homing Endonucleases

### Phage genomes as hosts for homing endonucleases

Phage T4 is an oddity among T-even phage, encoding 15 homing endonucleases. We pondered the significance of this observation in 2000, suggesting that the number of completely sequenced T-even phage genomes was too few to say anything definitive about endonuclease distribution [75]. In the intervening years, many more phage genomes have been sequenced [47,76-80], yet the trend holds - T4 remains the outlier, as most genomes have few endonuclease insertions. Based on these observations, it is tempting to conclude that there exists significant evolutionary pressure for phage to resist colonization by homing endonucleases. This conclusion, however, is at odds with the *in vitro *and *in vivo *characterization of endonuclease-mobility pathways that rely on extremely efficient DNA repair and recombination pathways to promote dissemination of homing endonucleases through populations of phage lacking them. Our understanding of factors that influence endonuclease mobility and retention in phage genomes is still in its infancy, but studies with eukaryotic homing endonuclease systems may provide some clues. In particular, studies on intron-encoded yeast homing endonucleases have elucidated a cyclical life cycle that allows homing endonuclease genes to escape degeneration and deletion due to lack of intron-less alleles for homing [81,82]. Related endonuclease life cycles have been proposed for transposition of homing endonucleases to new sites within a phage genome, allowing the endonuclease to escape deletion [41,83]. Moreover, recent modeling studies suggests that homing endonuclease genes could persist for significant time frames in the absence of homing sites and selection for a functional homing endonuclease [84].

One unifying characteristic of phage-encoded homing endonucleases is the observation that most endonuclease genes are inserted within or near phage genes that are functionally critical, such as DNA polymerases and ribonucleotide reductases. Targeting of functionally critical phage genes by homing endonucleases is an evolutionary strategy to maximize spread because very similar genes and target sites will be present in related genomes [75], and to minimize loss (see below). Moreover, the recognition sites of many homing endonucleases often encompass nucleotide sequence that corresponds to functionally critical amino acid (or RNA) residues of the host gene, often encoding an active site or essential region of the host gene [85,86]. It is also the case that homing endonucleases of different classes will target the same gene. For instance, the *nrdB *gene encoding the small subunit of aerobic ribonucleotide reductase is cleaved by both *mobE*, an HNH endonuclease of phage T4 [42], and by a unique endonuclease, *hef *(homing endonuclease-like function), encoded in phage U5 [41]. Similarly, the anaerobic ribonucleotide reductase proteins, encoded by the *nrdD *and *nrdG *genes, are targeted by *seg *and *mob *homing endonucleases [41]. Given the relatively small size of phage genomes, and the fact that many phage genes are essential, it is not surprising that similar target sequences have independently been selected as recognition and cleavage sites by different classes of homing endonucleases.

The insertion sites of many self-splicing group I introns also correspond to functionally critical sequences in phage genomes. Insertion of the intron into a functionally critical region is thought to prevent deletion of the element from the phage genome, as only a precise deletion of the intron or intein will restore a functional host gene sequence, whereas an imprecise deletion would likely be lethal. The propensity for homing endonucleases and introns to target conserved sequences forms the core of a recently proposed evolutionary scenario termed collaborative homing, for the origin of mobile introns by recombination between an endonuclease-lacking intron and a free-standing endonuclease that is "pre-adapted" to target the intron insertion site of the endonuclease-lacking intron, creating a highly efficient composite mobile genetic element [87,88]. The very similar *trans *homing pathway involves a free-standing homing endonuclase, *mobE*, mobilizing the defunct I-TevIII endonuclease and *nrdB *intron in phage T4 [42].

### Impact of homing endonucleases on phage genome structure and function

Because homing endonucleases utilize DNA repair and recombination pathways to promote mobility, significant co-conversion of sequence flanking the endonuclease's cleavage site sequence is associated with endonuclease-mediated mobility [14,18,39]. This observation helps explain a long-known phenomenon of T-even phage biology, namely the exclusion of T2 markers from progeny of a T2 and T4 coinfection [89]. Marker exclusion was first described in 1974, but it was not until almost 30 years later that a link between homing endonucleases and marker exclusion was uncovered [35,36]. Strikingly, the recognition and cleavage sites of many characterized T4-encoded homing endonucleases correspond to sites in the T2 genome that are excluded from the progeny of a T2 and T4 coinfection. Cleavage of T2 by a T4-encoded endonuclease initiates a localized gene conversion event at the cleavage site that replaces T2 with T4 sequence, resulting in the exclusion of T2 markers from progeny. A similar marker exclusion phenomenon involving intron-encoded endonucleases was also observed in HMU phage of *Bacillus subtilis *[43,90]. Thus, homing endonucleases influence the distribution of sequences flanking their insertion site within populations of related phage, in essence promoting lateral gene transfer.

More dramatic effects on phage gene structure and function arise from what appear to be homing endonuclease transposition events, whereby an endonuclease gene has inserted into a site that is different from the insertion site of analogous homing endonucleases in related phage genomes. Such transposition-like insertions include the *mobE *insertion into *nrdA *large subunit gene of aerobic ribonucleotide reductase of *Aeromonas hydrophila *phage Aeh1 [91], the *mobA *insertion into the topoisomerase large subunit gene *60 *of phage T4 [47], and a *seg43(25) *insertion associated with gene *43*, encoding a B-type DNA polymerase of *Aeromonas *phages 25 [88]. In the *mobE *and *mobA *cases, the homing endonuclease has inserted into a functionally critical region of the host gene, splitting the gene into separate coding regions as compared to related phage. For the *seg*-like insertion of phage 25, it is difficult to ascertain whether the split gene *43 *structure arose by insertion of the *seg *homing endonuclease, because the related phage 44RR possess a different genetic arrangement, consisting of an intercistronic untranslated sequence (IC-UTS) that splits gene *43 *into *43A *and *43B *[88]. Thus it is possible that the *seg *endonuclease invaded an already split gene created by the insertion of the IC-UTS.

Regardless of their origins, each insertion splits a contiguous coding region into two distinct polypeptides that must somehow reassemble to form a functional enzyme. Recent work has shown that the split *nrdA *gene of phage Aeh1 encodes a fully functional aerobic ribonucleotide reductase with activity similar to canonical enzymes that consist of a single NrdA polypeptide [91]. Similarly, the split *43A *and *43B *genes of phage 25 co-purify when overexpressed, and possess DNA polymerase activity [88]. Although the *mobA *insertion has not been studied in detail, phage T4 topoisomerase has long served as a model enzyme and possesses an unusual subunit structure with respect to other phage-encoded and bacterial topoisomerases [92], consistent with assembly of the split topoisomerase polypeptides to form a functional enzyme. How the split polypeptides assemble to form functional complexes in each of the enzyme systems is a fascinating structure and function question.

## Conclusion

T-even phage have proven to be an attractive and tractable model system for studying the biology of homing endonucleases in the last 20 years, and we have learned much about the molecular details of mobility pathways and regulatory mechanisms. Many of these details are applicable to homing endonucleases in eukaryotic systems, and also have provided insight into the mobility pathways of other mobile elements such as inteins and group II introns. From a mechanistic perspective, how the *mob *endonucleases spread between genomes by nicking their target sites rather than introducing a double-strand break is an intriguing area of future research. From a genomic perspective, the relatively small size of phage genomes coupled with extraordinary advances in sequencing technology has revealed that homing endonuclease genes are widespread, but not as abundant as predicted based on laboratory experiments. It remains to be determined if more phage genome sequences can offer insight into evolutionary processes that regulate homing endonuclease distribution, as it already clear from existing sequences that complex regulatory mechanisms have evolved to control the expression of homing endonucleases. Clearly, there are interesting evolutionary forces at work, and experimentally manipulating regulatory controls will likely be required to understand the impact of homing endonuclease activity on phage genome structure and function.

## Competing interests

The authors declare that they have no competing interests.

## Authors' contributions

DRE, EAG and MB wrote the manuscript. All authors read and approved the final manuscript.
